# Integration of Specific Aeration Demand (SAD) into Flux-Step Test for Submerged Membrane Bioreactor

**DOI:** 10.3390/membranes15040111

**Published:** 2025-04-03

**Authors:** Albert Galizia, Joaquim Comas, Ignasi Rodríguez-Roda, Gaëtan Blandin, Hèctor Monclús

**Affiliations:** 1 LEQUIA, Institute of the Environment, Universitat de Girona, C/Maria Aurèlia Capmany 69, 17003 Girona, Catalonia, Spain; albert.galizia@udg.edu (A.G.); joaquim.comas@udg.edu (J.C.); ignasi.rodriguezroda@udg.edu (I.R.-R.); gaetan.blandin@udg.edu (G.B.); 2 Catalan Institute for Water Research (ICRA), C/Emili Grahit 101, 17003 Girona, Catalonia, Spain

**Keywords:** MBR, SADm, operational conditions, UF-HF

## Abstract

This study proposes a novel methodology to assess fouling that complements the flux-step test (FST) by integrating aeration-step tests (ASTs) to optimise the specific aeration demand (SADm) for ultrafiltration hollow-fibre (UF-HF) submerged membranes in membrane bioreactor (MBR) configurations. Three membranes with distinct manufacturing processes—non-thermal-induced phase separation (NIPS) and thermal-induced phase separation (TIPS)—were evaluated under continuous and intermittent aeration. The AST revealed that the critical SADm has a range of 0.1–0.5 m^3^·m^−2^·h^−1^ for continuous aeration and 0.1–0.2 m^3^·m^−2^·h^−1^ for intermittent aeration. NIPS membranes with homogeneous structures were less prone to fouling under intermittent aeration, while TIPS membranes with a heterogeneous structure exhibited better recovery under continuous aeration, reflecting distinct fouling dynamics. Findings indicate that the FST alone does not fully represent operational conditions, as aeration efficiency is linked to membrane structure and aeration mode. By combining the FST with ASTs, our approach enables tailored fouling control strategies, reducing energy consumption and improving MBR performance. These insights are critical for advancing toward energy-efficient wastewater treatment technologies.

## 1. Introduction

Membrane bioreactors (MBRs) are a well-established technology in wastewater treatment plants (WWTPs) that produce high-quality effluent compared to conventional activated sludge (CAS) systems [1]. The primary advantage of MBR technology is its ability to deliver superior effluent quality. MBRs reduce the concentrations of suspended solids, bacteria, viruses, and emerging pollutants in WWTP effluent much more effectively than CAS systems [2,3,4,5]. Despite these advantages, MBRs are associated with higher capital expenditures (CAPEX) and operational expenditures (OPEX) compared to CAS systems. Among OPEX, energy consumption required for membrane aeration is particularly significant, accounting for up to 50% of the total energy demand of the WWTP [6,7]. As a result, optimising aeration processes can lead to significant energy savings in WWTPs [8].

Another critical challenge is membrane fouling, which compromises performance and reduces the operational lifespan of the membranes. Fouling is a multifaceted phenomenon driven by factors such as membrane pore blocking and cake layer formation, both of which result in decreased permeate flow and reduced permeability during filtration [9].

Membrane fouling can be categorised into three types based on removability: reversible fouling, which can be managed through physical cleaning actions; irreversible fouling, which requires chemical cleaning to restore filtration performance; and permanent fouling, which is permanent and linked to the membrane’s lifespan [9,10].

The fouling control strategy for each process is influenced by membrane type, water complexity, and reactor configuration (submerged or side-stream). Membrane types are typically classified by configuration [e.g., flat-sheet (FS), hollow-fibre (HF), multi-tubular (MT)], pore size [e.g., microfiltration (MF), ultrafiltration (UF), nanofiltration (NF)], and material [e.g., polyethylene (PE), polyvinylidene fluoride (PVDF), porous polytetrafluoroethylene (PPTFE), etc.] [11]. Strategies are usually aimed at managing reversible fouling, which occurs during short-term operation but can lead to more severe fouling if not controlled. The inadequate management of reversible fouling can accelerate the onset of irreversible fouling, thereby limiting long-term membrane performance and increasing the chemical cleaning demand.

Fouling control strategies vary according to the membrane classification. Pore size is a key factor influencing the dominant fouling mechanisms and the most effective control methods. MF) and UF membranes, with larger pores, are more prone to cake layer formation and concentration polarisation. In these cases, hydrodynamic strategies such as optimised crossflow velocity, aeration, and relaxation cycles are commonly used to reduce fouling. For severe fouling, like pore blocking and adsorption, these membranes require additional strategies such as backwashing, chemical cleaning, and surface modifications to maintain performance. In all cases, a combination of preventive (e.g., optimised operational parameters [8], hydrodynamic control) and corrective (e.g., physical or chemical cleaning) strategies is necessary to ensure membrane longevity and minimise performance decline [12].

In addition to these conventional methods, alternative fouling control strategies have been investigated, including quorum quenching (QQ), enzymatic treatments, and nanomaterial-based coatings. These approaches aim to mitigate biofouling by disrupting microbial communication, degrading extracellular polymeric substances (EPSs), or modifying membrane surface properties. However, despite promising results at the laboratory and pilot scale, their application in full-scale plants remains limited due to challenges related to cost, scalability, and long-term stability. Further research is required to optimise these emerging strategies and facilitate their integration into large-scale membrane bioreactor (MBR) systems [13].

Reversible fouling can be mitigated with physical control actions such as membrane aeration, relaxation, backwashing, or reducing operational flux (J), thus maintaining the operational flux under critical conditions [14]. Critical flux (Jc) is defined as the flux at which reversible fouling can be controlled through physical corrective actions [15]. In typical MBR operations, these corrective actions are grounded on an operational cycle that includes filtration followed by relaxation or backwashing, with continuous or intermittent membrane aeration. Most submerged membrane processes use relaxation and aeration as primary strategies to control reversible fouling in wastewater treatment [16].

Jc is usually specified by manufacturers, but it can also be determined in lab-scale conditions using methods such as the flux-step test (FST) [17]. In the FST, a continuous, high, and constant aeration flow rate is applied with alternating periods of filtration and relaxation, while flux (J) is gradually increased. Fouling is monitored through transmembrane pressure (TMP), with Jc identified as the highest flux at which the TMP or fouling rate (FR) (dTMP/dt) remains stable, indicating that physical cleaning actions (aeration and relaxation/backwashing) are sufficient to control reversible fouling. Additionally, Monclus et al. (2010) [18] quantified a FR threshold of 0.5 mbar·min^−1^ for UF-HF membranes to differentiate between critical and subcritical conditions. However, full-scale facilities do not typically operate at the high aeration rates used in lab-based Jc determination, which limits the direct applicability of this method. Additionally, there is currently no evidence of Jc determination being widely implemented in full-scale WWTPs.

Membrane aeration flow rates are often expressed as the specific aeration demand (SAD), which can be based on the membrane surface area (SADm, m^3^ air·m^−2^·h^−1^) or on permeate flow rate (SADp, m^3^ air·m^−3^ permeate). Manufacturers typically specify cyclic operations, including filtration and relaxation/backwashing cycles, with a recommended SADm between 0.5 and 1.5 m^3^·m^−2^·h^−1^ [16]. These recommended SADm are generally conservative, designed to ensure maximum membrane cleaning and minimise fouling risks; however, they often do not account for potential energy optimisation. The aeration mode may be continuous or intermittent, depending on plant management, membrane configuration, and manufacturer guidelines. Commonly, SADm values are determined based on air scour flow rate manufacturer recommendations, without considering the external structure of the membrane. There are two main techniques for manufacturing UF membranes for MBRs: thermal-induced phase inversion separation (TIPS) and nonsolvent-induced phase inversion separation (NIPS). These techniques produce membranes with distinct external structures, which may have different fouling dynamics [19,20,21,22]. This variability suggests that aeration strategies may need to be tailored according to the membrane’s external structure to optimise fouling control [23].

Considering all aspects explained above, membrane fouling can be influenced by many factors, including the membrane external structure, membrane permeate flux (J), membrane aeration flow rate, or even the membrane aeration strategy; therefore, it is still necessary to develop new strategies to help understand fouling phenomena and support fouling mitigation.

The primary objective of this study was to introduce a complementary methodology to improve the critical flux (Jc) evaluation method by combining the Jc estimation with the effect of the air scour flow rates to determine a critical SADm (SADmc) for UF-HF membranes. A secondary objective was to evaluate the impact of the aeration strategy on different UF-HF membranes with different external structures to determine how these differences influence fouling behaviour and mitigation.

## 2. Materials and Methods

This section is divided into four subsections: (1) the description of the pilot plant setup, (2) the characterisation methods for membranes, including both physical and operational characteristics, (3) the characterisation of the sludge used in the experiments, and (4) the aeration-step test for the determination of the critical specific aeration demand per membrane surface area (SADmc).

### 2.1. Pilot Plant Setup

**Figure 1:**
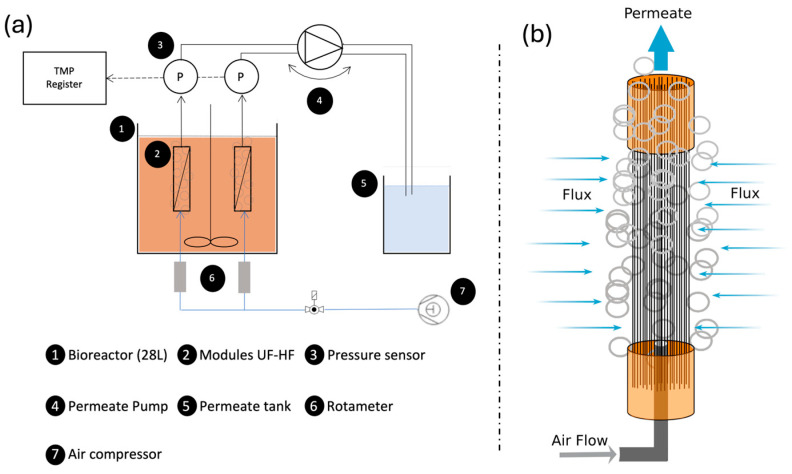
(**a**) Setup diagram; (**b**) UF-HF module diagram.

This study was conducted using a lab-scale pilot plant with a 28 L reactor, designed to operate up to two membrane modules in parallel, filtering from outside to inside, as typically configured in MBR systems. Each has an independent air scour flow rate, allowing for precise control over the air scour flow rates to assess their impact on membrane fouling (Figure 1a). Thus, the pilot plant allows us to test the membrane modules simultaneously, providing the flexibility to compare performance under different operational conditions under the same sludge environment. Each module is equipped with an independent system for monitoring the TMP and separating permeate pumps to control the permeate flow rate for each module.

The modules used in the reactor were self-constructed and comprised 25 to 30 HF membranes with lengths ranging from 210 to 250 mm (Figure 1b). The number of fibres and their lengths were adjusted according to the external diameter of each membrane type, ensuring they fit properly within the reactor and provide a similar filtration surface area for each module.

The setup was operated using real activated sludge sourced from an urban wastewater treatment plant (WWTP) located in Quart (Catalonia, NE of Spain), simulating realistic operational conditions for the evaluation of membrane performance.

### 2.2. Sludge Characterisation

To evaluate the operational conditions for each test, various characterisation assays were performed using samples of the biological sludge from the reactor. The assays included measurements of total suspended solids (TSSs), filterability, and capillarity suction time (CST). The TSS was measured according to the APHA 2540c method, which involved filtering a known volume of sludge through a filter with a diameter of 47 mm and a pore size of 1.5 µm. The filtered sludge was then dried in an oven at 105 °C for 24 h, and the TSS was determined by measuring the difference in weight before and after drying. Filterability was assessed using a 50 mL sample of sludge, with the filtered volume measured after 5 min using 185 mm filter paper, following the procedure described by Rosenberger and Kraume (2002) [24]. The CST was measured according to the methodology outlined by Scholz (2005) [25] using specific CST laboratory equipment. During all tests, the sludge characteristics were similar, with a TSS of 6 ± 1 g·L^−1^, a filterability of 28 ± 3 mL, and a CST of 30 ± 5 s.

### 2.3. Membrane Characterisation

The membranes used in this study were acquired from three different manufacturers: Polymem^®^, Castanet-Tolosan, France; Scinor^®^, New York, NY, USA; Scinor^®^ and Mitsubishi^®^, Tokyo, Japan. All membranes were designed for wastewater treatment applications in membrane bioreactor (MBR) processes. According to the manufacturers, the membranes were fabricated using distinct production methods, and each type exhibited varying external diameters (De) (Table 1).

The characterisation of the membranes included both physical and operational aspects. Physical characteristics such as membrane diameters, wall structure, and module surface area were obtained from manufacturer specifications and validated using scanning electron microscopy (SEM). The SEM analyses were performed using a field-free analytical UHR SEM, TESCAN CLARA model. Membrane samples were first submerged in liquid nitrogen and then cut with a blade to produce clean cross-sectional cuts. The prepared membrane sections were mounted on sample holders and coated with a silver alloy to minimise charging effects and offer excellent charge dissipation. Subsequently, the samples were covered with a thin layer of carbon to ensure surface conductivity. Images were acquired at an accelerating voltage of 5 keV and a working distance (WD) of approximately 27 mm. Various magnifications were used: 200× to observe the internal and external diameters (Di and De) of the membranes, 1000× to examine the membrane wall, and 2500× to assess the homogeneity of the external membrane layer. These magnifications were chosen to provide a comprehensive view of the membranes at different scales, highlighting their overall structure, detailed wall composition, and external surface characteristics.

The membranes’ operational characterisations included measurements of liquid permeability (LP) and critical flux (Jc); all experiments were performed at constant and controlled room temperature of 22 °C. LP tests were conducted in the setup (Figure 1a) using pure water. During the test, TMP was monitored to obtain near-continuous readings, with measurements taken every 5 s. The test began at a low operational J, ensuring that TMP values remained at low values (below 0.1 bar). Flux (J) was progressively increased while continuously monitoring TMP. This process continued until sufficient data points were collected to calculate membrane permeability (K), expressed in LMH·bar^−1^, by determining the slope of the TMP versus J plot.

The estimation of Jc was performed using the classical flux-step (FS) method [17,26,27]. In this study, the specific protocol involved maintaining a high and constant SADm, conducting filtration cycles of nine minutes followed by one minute of relaxation, and incrementally increasing the J by 5 LMH every two filtration cycles. Each cycle was replicated to obtain averaged fouling rates (FRs), which were calculated as the slope of TMP versus time during the filtration phase of each cycle. The experiment was terminated once a TMP limit of 500 mbar was reached. The Jc was determined as the maximum flux at which the FR remained below the threshold of 0.5 mbar·min^−1^, in line with the criteria established in [28]. To prevent sludge concentration during the test, the permeate flow was recirculated to the reactor without adding any substrate. The reactor’s level and sludge concentration were kept constant.

### 2.4. Aeration-Step Test (AST) Methodology

The proposed novel methodology to determine the critical specific aeration demand per membrane surface area (SADmc) combines elements from the flux-step test (FST) and the aeration-step test (AST) described by Monclus et al. (2010) [18]. During the AST, the permeate J was maintained constant, set just below the Jc as determined from a previous FST. Flux consistency was verified at the beginning, midpoint, and end of the tests. Membranes were operated in cycles of 9 min of filtration followed by 1 min of relaxation, without backwashing. Aeration started at the highest SADm value selected for each specific test, which varied depending on the experiment. Then, this value was progressively reduced in five steps until reaching zero. The maximum SADm values tested are detailed in the Results section. Once zero was reached, the SADm was then incrementally increased back to the initial value, following the same steps in reverse. Tests were conducted both under continuous and intermittent aeration modes. Intermittent aeration was controlled by electro valves operated by a PLC, alternating on and off every 5 s. The fouling rates (FRs) were analysed for each filtration cycle to determine the SADmc. The critical SADm was identified as the aeration rate at which the FR remained below the threshold of 0.5 mbar·min^−1^, consistent with the criteria used for Jc determination. To prevent sludge concentration during the test, the permeate flow was recirculated to the reactor without adding any substrate. The reactor’s level and sludge concentration were kept constant.

## 3. Results

The results of this study are divided into two main subsections: (1) membrane characterisation and (2) the determination of the critical specific aeration demand per membrane surface area (SADmc) for each membrane.

### 3.1. Membrane Characterisation

#### 3.1.1. SEM Analysis

**Figure 2:**
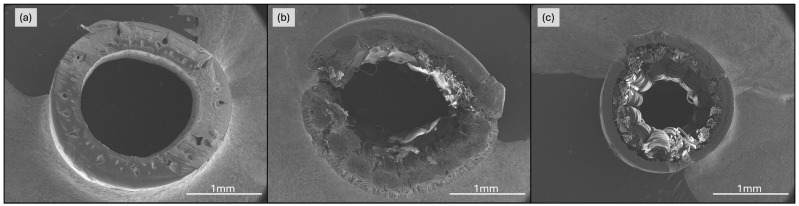
SEM images from membrane wall structure at 200×: (**a**) M1 membrane; (**b**) M2 membrane; (**c**) M3 membrane. Detailed images at 1000× and 2500× can be found in the Supplementary Materials.

The structural differences between the M1, M2, and M3 membranes revealed distinct visual and structural characteristics for each membrane type. The M1 membrane demonstrated a uniform and homogeneous wall structure in both its external and internal layers (Figure 2a). The external surface appeared uniform, with consistently distributed pores that were evenly spaced throughout the membrane, indicating a homogenous formation process. This structure aligns with the typical characteristics of NIPS synthesised membranes.

In contrast, M2 membrane exhibited a more complex structure. The external layer featured heterogeneous pores with a finger-like macroporous pattern on the outer surface. These macropores were significantly larger and more irregular compared to those of the M1 membrane, a characteristic consistent with membranes fabricated using the TIPS technique. Beneath the external layer, an intermediate layer with higher density was observed, followed by an internal layer with a textile-like structure that provides additional mechanical strength (Figure 2b). Although less porous, this internal layer played a crucial role in the overall structural integrity of the membrane, as indicated by the manufacturer.

The M3 membrane featured a combination of the structures observed in M1 and M2 with (1) an homogenous external surface similar to that of the M1 membrane and typical of membranes fabricated using the NIPS technique; and (2) its internal structure was like that of the M2 membrane, heterogeneous and dense with a textile-like composition, designed to provide additional mechanical support (Figure 2c). This dual structure reflects a balance between external uniformity and internal strength, combining the benefits of both manufacturing approaches.

UF-HF modules were constructed using each of the studied membranes. Once constructed, integrity tests were performed, and the total filtration surface area of each module was determined (Table 2).

After ensuring the integrity of each module and calculating their total filtration surface areas, characterisation tests for liquid permeability (LP) and flux-step (FS) were conducted to evaluate the filtration capacity of each module.

#### 3.1.2. Liquid Permeability (LP)

**Figure 3:**
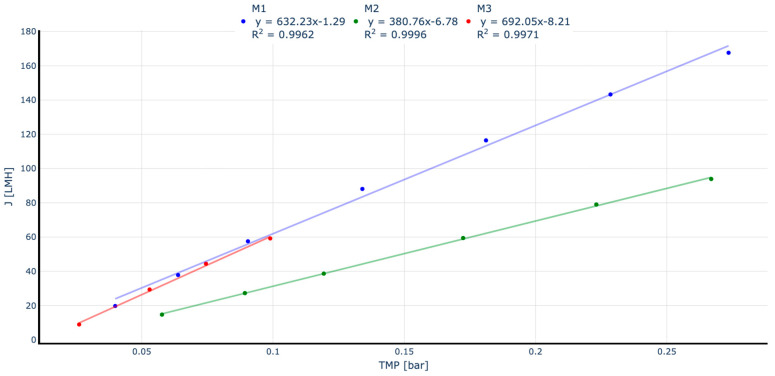
Liquid permeability results for each manufacturer module.

The liquid permeability (LP) for the three membranes demonstrates a clear linear relationship between J and TMP, with an R^2^ value exceeding 0.99. This indicates linear performance, confirms membrane structural integrity, and provides an initial performance comparison (Figure 3).

The ratio between J and TMP, known as permeability (K) and typically expressed in LMH·bar^−1^, reflects the membrane’s ability to facilitate water permeation. Higher permeability values indicate a greater capacity to produce permeate with lower energy requirements. While all tested membranes exhibited linear permeability (K), notable differences were observed. Membranes M1 and M3, both with homogeneous external surfaces, displayed similar permeability values of approximately 600 LMH·bar^−1^. In contrast, M2, characterised by macropores on its external surface, showed a significantly lower permeability of under 400 LMH·bar^−1^, approximately 33% less than the other two membranes. These findings suggest that membranes with micropores and a homogeneous external structure achieve superior performance in permeate production. After completing the LP tests, a critical flux-step test was conducted to estimate the critical flux under MBR operating conditions using real wastewater treatment activated sludge.

#### 3.1.3. Critical Flux (Jc) Estimation

The critical flux (Jc) was determined using the flux-step test (FST) in a lab-scale setup with real WWTP activated sludge. During the test, the membranes operated under continuous aeration with a constant specific aeration demand per membrane surface area (SADm) of 1.5 Nm^3^·m^−2^·h^−1^, following a typical filtration cycle as outlined in the methodology.

**Figure 4:**
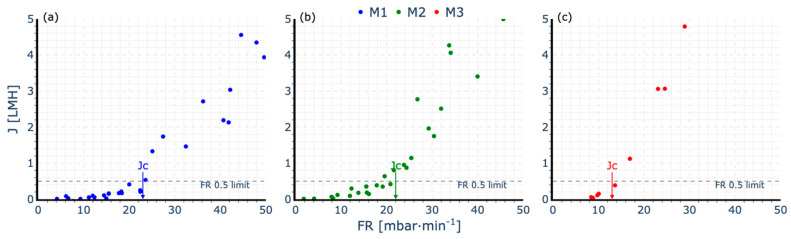
Jc estimation for each membrane: (**a**) M1 module; (**b**) M2 module; (**c**) M3 module.

In monitoring the fouling rate (FR) and applying a threshold of 0.5 mbar·min^−1^, the Jc was estimated for each membrane. The M1 membrane exhibited a critical flux of approximately 23 LMH (Figure 4a), indicating stable filtration performance before the onset of significant fouling. Similarly, the M2 membrane reached a Jc of 22 LMH (Figure 4b), showing that despite its lower permeability, it could sustain a comparable critical flux when operating with continuous aeration and a high SADm. In contrast, the M3 membrane showed a significantly lower critical flux of 12 LMH (Figure 4c), suggesting that despite its higher permeability, it is more prone to fouling under identical operational conditions, reaching its fouling threshold at a lower flux compared to the other membranes.

The FST results suggest that under conditions of high SADm and continuous aeration, M3 exhibits poorer performance despite having physical characteristics that combine features from both M1 and M2.

### 3.2. Aeration-Step Test (AST)

For this study, two types of ASTs were designed: a partial AST and a full AST. The partial AST involves only the gradual reduction in the aeration flow rate, while the full AST incorporates both the reduction and subsequent increase in the aeration flow rate. These tests were conducted to evaluate the impact of aeration strategies on membrane fouling under controlled conditions.

#### 3.2.1. Partial AST

To evaluate the effect of aeration on membrane performance, two partial ASTs were conducted: one at half the Jc and another at double the Jc. Both tests utilised continuous aeration, starting at a high specific SADm, as in the FST methodology. The aeration flow rate was then progressively reduced to zero to assess its impact on the FR.

**Figure 5:**
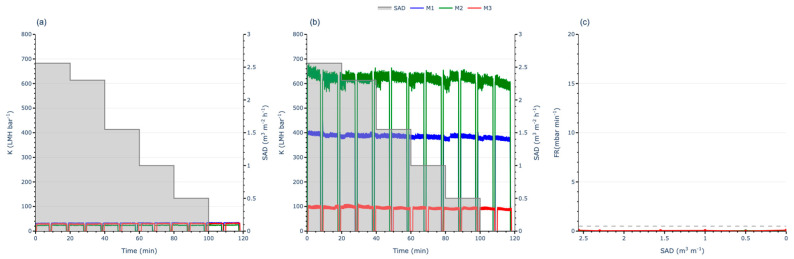
Partial AST working at half of Jc for each membrane: (**a**) TMP evolution; (**b**) permeability evolution; (**c**) FR evolution.

Working at low J (half of Jc), no significant increases in TMP or losses in K were observed (Figure 5a,b). Additionally, the FR remained below the threshold throughout the test (Figure 5c), regardless of whether aeration was applied. This indicates that no fouling phenomena occurred at low permeate flux, as there were no differences in membrane performance between operating with or without aeration. This phenomenon suggests that the aeration flow rate applied during the initial steps of the FST may not have influenced the results. Moreover, this behaviour aligns with the recommendations of membrane manufacturers, who typically suggest operating close to the Jc to avoid oversized surface modules and minimise energy consumption.

**Figure 6:**
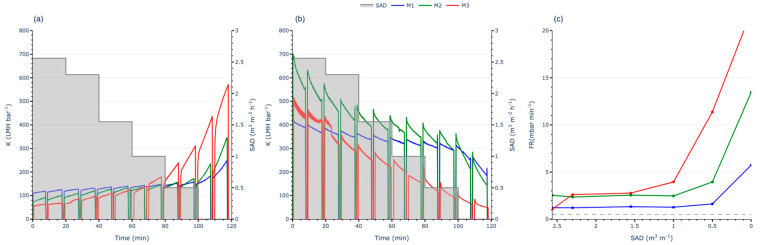
Partial AST working at double the Jc for each membrane: (**a**) TMP evolution; (**b**) permeability evolution; (**c**) FR evolution.

In contrast, when operating at high J (double the Jc), a clear increase in TMP and a decrease in K were observed as aeration decreases (Figure 6a,b). This resulted in the FR exceeding the threshold even at the first step (Figure 6c), where the SADm was at its maximum. These findings indicate that significant fouling phenomena occurred on the membrane due to the high flux conditions, despite the high aeration applied. In the initial steps of the AST, it can be observed that even though the FR exceeded the threshold, the TMP slope remained relatively stable, suggesting that the high aeration rate effectively mitigates reversible fouling. However, as the aeration rate decreased, the FR began to increase sharply, indicating that the reduced aeration flow rate was no longer sufficient to manage the reversible fouling. With each subsequent step, fouling increases, reaching the operational limit of 500 mbar for M3 and causing a loss of over 80% of its K.

Differences emerged among the three membranes: M1 demonstrated lower sensitivity to aeration intensity, while M3 was highly sensitive. These findings underscore the importance of SADm intensity when operating above critical flux. Comparing these observations with those recorded under sub-critical flux conditions reveals key behaviours essential for overall MBR optimisation. This indicates that optimising only the permeate flux is insufficient. Effective fouling control necessitates the joint optimisation of J and SADm.

This highlights the limited effectiveness of physical corrective actions, such as aeration, under extreme operating conditions. At low fluxes, fouling is minimal and does not require significant corrective measures in the short term. However, at high fluxes above the Jc, fouling becomes severe and demands a high SADm to mitigate reversible fouling. Even then, a high SADm may not always suffice to maintain subcritical conditions, leading to a continuous decline in K. Under these circumstances, the FR increases rapidly, even at the highest aeration flow rates, confirming that fouling becomes unmanageable when operating beyond the Jc. These results also indicate that the FST methodology may not fully capture the operational conditions required to determine Jc accurately, reinforcing the importance of linking Jc to the SADm under which the membrane operates.

#### 3.2.2. Full AST

Full ASTs were conducted at a permeate flux close to (but below) the Jc for each membrane, as this is the range where aeration regulation has the greatest influence on fouling. Previous tests demonstrated that, at very low fluxes, aeration has little to no visible effect on the FR. Conversely, at high fluxes (twice the Jc), aeration requirements become significantly higher, and fouling becomes severe. By operating near the Jc, it is possible to better assess the impact of aeration strategies, balancing fouling control and aeration intensity. Two types of aeration strategies were assessed: continuous aeration and intermittent aeration. These tests enabled a detailed analysis of the effect of different aeration modes on fouling rates, reflecting typical aeration strategies employed in full-scale plants.

#### 3.2.3. Full AST with Continuous Aeration

In these tests, the initial SADm was set at 1.3 Nm^3^·m^−2^·h^−1^. The SADm was progressively reduced to zero in five steps and then incrementally increased back to 1.3 Nm^3^·m^−2^·h^−1^. Throughout the process, TMP was monitored, and the J was checked to ensure its constancy; then, K and the FR were calculated to evaluate the membrane performance.

**Figure 7:**
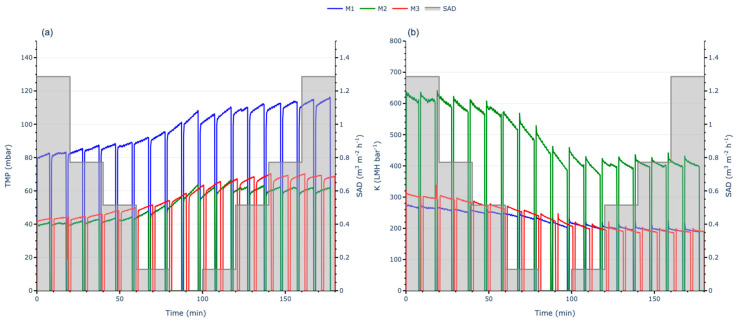
Full AST with continuous aeration results: (**a**) TMP evolution; (**b**) K evolution.

The results highlight distinct behaviours among the membranes. The M1 membrane initially operated at higher TMP values compared to M2 and M3, despite M1 and M3 sharing a similar NIPS external structure (Figure 7a). At the start of the test, M1 registered a TMP of approximately 80 mbar, nearly double that of M2 and M3, which both began at around 40 mbar. M2 started with a lower TMP close to 40 mbar (Figure 7b).

In contrast, M1 and M3, which started with lower initial permeabilities, exhibited smaller losses during the test, with both showing a decrease in permeability from 300 to 200 LMH·bar^−1^ (approximately 33%). Despite the TMP for M1 being nearly double that of M3, their permeability trends were comparable, indicating similar performance for membranes with an external NIPS membrane structure (M1 and M3) under continuous aeration. Conversely, the TIPS membrane (M2) experienced the most significant permeability loss, dropping from 650 to 400 LMH·bar^−1^ (a 38% decline).

In all cases, permeability showed a clear and progressive decline as the SADm was reduced, reaching near-minimum levels when aeration was set to zero. Although increasing the aeration again mitigated further fouling development (TMP did not continue increasing), permeability was not recovered back to its initial levels, stabilising instead at reduced values. This behaviour indicates that irreversible fouling formed under the test conditions, which could not be fully alleviated by increased aeration alone. However, higher aeration flow rates were effective in controlling reversible fouling, preventing further irreversible fouling and additional performance loss. To validate these observations, statistical analyses were performed to determine whether the differences in permeability evolution were statistically significant.

To further assess the observed permeability trends, normality and variance comparison tests were conducted to determine whether significant differences existed between the curves. The Shapiro–Wilk and Kolmogorov–Smirnov tests indicated that none of the membranes met the assumption of normality. Consequently, the non-parametric Mann–Whitney U [29] test was performed to compare M1 with M2, M1 with M3, and M2 with M3. The test results yielded *p*-values < 0.05 for all comparisons, indicating statistically significant differences in permeability evolution among all membranes.

#### 3.2.4. Full AST with Intermittent Aeration

The aeration flow rate alternated between 5 s on and 5 s off while maintaining the same air scour flow rate (m^3^·h^−1^) during the “on” periods as in the continuous aeration test. On the other hand, SADm was also divided by two due to the intermittent aeration setup. The SADm started at 0.65 Nm^3^·m^−2^·h^−1^ and was progressively reduced to zero in five steps, and then incrementally increased back to 0.65 Nm^3^·m^−2^·h^−1^ following the same proportions. Throughout the process, TMP was monitored, and the J was checked to ensure its constancy; then K and the FR were calculated to evaluate the membrane performance.

**Figure 8:**
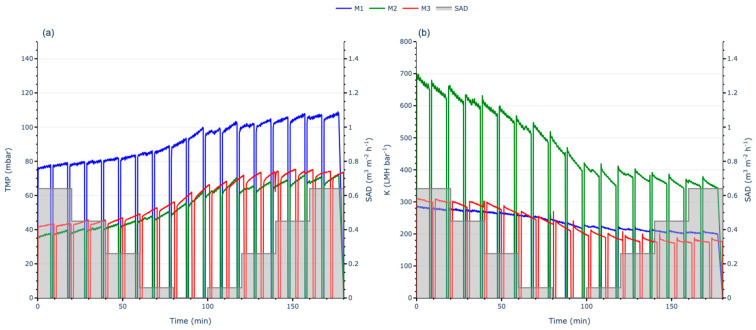
Full AST with intermittent aeration results: (**a**) TMP evolution; (**b**) K evolution.

The initial trends were like those observed in the full AST with continuous aeration. The M1 membrane operated with higher TMP, while the M2 and M3 started at lower TMP levels (Figure 8a). As in the previous test, M2 had the highest K throughout the test (Figure 8b).

However, with time and in decreasing the SADm, the M3 membrane experienced the greatest loss in permeability, decreasing from 320 to 200 LMH·bar^−1^, which corresponds to a permeability loss of approximately 38%. The M1 membrane showed a smaller decline, with permeability reducing from 280 to 220 LMH·bar^−1^, representing a loss of about 21%. The M2 membrane followed a similar trend, with permeability dropping from 700 to 480 LMH·bar^−1^, corresponding to a loss of approximately 31%. Following a similar pattern, membranes with a more homogeneous NIPS structure exhibited lower permeabilities throughout the test. Nevertheless, in this instance, the M1 membrane, characterised as 100% NIPS, demonstrated the smallest permeability loss, whereas the M3 membrane, with its dual-layer structure, exhibited the highest percentage loss in permeability. These differences may result from the change in the aeration pattern when shifting to intermittent mode.

The overall permeability behaviour was consistent with the trends observed in the continuous aeration test. A progressive decline in permeability was noted as the SADm was reduced to zero. Upon increasing the aeration, permeability stabilised but did not recover to its initial values. The variation in results between continuous and intermittent aeration suggests that the process of determining the Jc using the FST methodology might not be fully representative, as it appears to be influenced by both the membrane’s structural characteristics and the type of aeration applied.

To compare the permeability evolution among the membranes, the same statistical tests—Shapiro–Wilk, Kolmogorov–Smirnov, and Mann–Whitney U—were performed. As in the previous case, the results confirmed statistically significant differences in permeability evolution across all membranes.

### 3.3. Critical SADm Determination

Based on all previous presented results, it becomes key to determine the critical SADm (SADmc). In order to do so, it is necessary to analyse the FR for each filtration cycle, applying the same criteria as in the FST. The critical fouling rate threshold of 0.5 mbar·min^−1^ was used, based on the methodology described for the FST.

**Figure 9:**
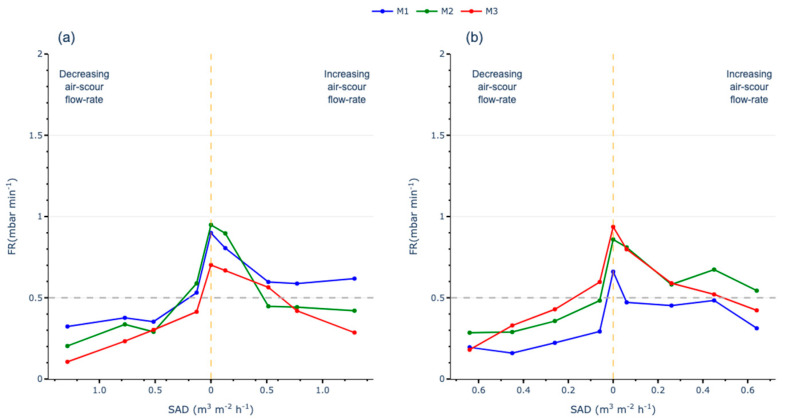
FR analysis for SADmc determination: (**a**) AST with continuous aeration; (**b**) AST with intermittent aeration.

In the full AST with continuous aeration, all membranes operated below critical conditions, even when the SADm approached its minimum. As the SADm was reduced to zero, the FR increased, eventually reaching critical conditions for all membranes. When the SADm was increased again, although neither TMP nor K recovered, the FR decreased, indicating that aeration mitigates fouling when working close to the Jc. Both M2 and M3 membranes returned to subcritical conditions during the increasing air scour flow rate phase, while M1, which experienced the least permeability loss, remained in critical conditions despite the reduction in FR (Figure 9a).

The results from the full AST with intermittent aeration show a similar trend to the continuous aeration test. All membranes initially operated under subcritical conditions until the SADm approached its minimum. The maximum FR was observed when the SADm was reduced to zero, where all membranes were operating under over-critical conditions. Upon increasing aeration, only M1 was able to effectively mitigate fouling, returning to subcritical conditions when the SADm reached its maximum again. The M2 and M3 membranes also exhibited some recovery, with their performance nearing subcritical conditions (Figure 9b).

The recovery behaviour of the membranes after reducing the SADm to zero reveals significant differences based on their wall structure. The M1 membrane, characterised by a completely homogeneous and thick wall (NIPS), shows irreversible fouling likely caused by the clogging of micropores on its surface or within its internal layer. In contrast, the TIPS-type membrane (M2), with a heterogeneous wall and macropores, accumulates more reversible fouling, allowing it to recover to subcritical conditions when the SADm is increased again.

The M3 membrane, which has a homogeneous external structure similar to M1 but a thinner wall, suggests that pore blockages are less profound compared to M1. This results in reversible fouling, which can be mitigated by increasing the aeration.

Conversely, under intermittent aeration, the M2 membrane remains in over-critical conditions, while the M1 and M3 membranes reduce their fouling. This behaviour may be attributed to the intermittent aeration generating momentary turbulence that disrupts preferential air pathways. In the case of NIPS membranes (M1 and M3), this turbulence likely prevents deep clogging, resulting in better fouling mitigation. Under intermittent aeration, the M3 membrane behaves similarly to M1. Due to its similar external structure, it experiences similar fouling patterns, avoiding the pore-clogging issues observed in the macropores of the M2 membrane.

Based on the results, the critical specific aeration demand (SADmc) for the membranes was established between 0.1 and 0.5 m^3^·m^−2^·h^−1^ under continuous aeration, provided the membranes are operating under subcritical conditions. Conversely, the SADmc can be set to ensure the membranes are operating with a permeate flux under subcritical conditions. Conversely, the SADmc can be reduced to a range of 0.1 to 0.2 m^3^·m^−2^·h^−1^ under intermittent aeration, narrowing the optimal operating range but potentially offering savings in aeration.

However, if fouling conditions have been already achieved and corrective actions are required, the SADmc increases significantly, reaching up to 1 m^3^·m^−2^·h^−1^ when using continuous aeration or up to 0.5 m^3^·m^−2^·h^−1^ under intermittent aeration. In such cases, it is important to note that aeration alone may not suffice, and additional corrective measures, such as reducing the J or applying chemical cleaning, may be necessary.

## 4. Conclusions

This study presents a methodology complementary to the flux-step test (FST) that provides valuable information on the optimal specific aeration demand (SADm) under different operational scenarios. While the FST remains a useful tool for estimating the critical flux (Jc), it has some limitations related to the real operational conditions or membrane intrinsic structure. The aeration applied during the FST proved to be largely irrelevant at low fluxes, when fouling was minimal and unaffected by aeration, while at fluxes above the Jc, aeration was insufficient to mitigate fouling. These findings highlight the need for additional methodologies, such as the aeration-step test (AST), to better capture the relationship between aeration and fouling control.

The results suggest a clear correlation between membrane performance and their structural characteristics. Membranes with a more homogeneous structure, typically manufactured using the NIPS process, demonstrated better performance under intermittent aeration. In contrast, membranes with a more heterogeneous external surface, associated with the TIPS manufacturing process, exhibited lower fouling rates under continuous aeration. This highlights the importance of considering membrane structure when optimising aeration strategies for fouling mitigation.

However, this differentiation between membrane structures was not observed in the determination of Jc, as membranes with different structures achieved similar critical flux values. While the FST provides a baseline for evaluating membrane performance, the AST is essential for determining the optimal SADm and tailoring aeration strategies to maximise efficiency.

Although this study was conducted in a lab-scale setup and may not be directly transferable to full-scale MBRs, our results can still contribute to optimising operational ranges in large- and full-scale applications. Through the integration of these methodologies into operational practices, it is possible to achieve optimised fouling control, reduced energy consumption, and improved long-term stability in membrane bioreactors.

## Figures and Tables

**Table 1:** Membranes’ physical characteristics.

Manufacturer	Alias	Fabrication Mode	TIPE	Material	De [mm]
Polymem^®^	M1	NIPS	HF-UF	PVDF	2.45
Scinor^®^	M2	TIPS	HF-UF	PVDF	2.60
Mitsubishi^®^	M3	Unspecified	HF-UF	PVDF	1.65

**Table 2:** Modules’ physical specifications.

Membrane	Structure	De [mm]	Length [mm]	N Fib	Surface [m^2^]
M1	NIPS	2.45	210	25	0.040
M2	TIPS	2.60	230	20	0.038
M3	Mix	1.65	250	30	0.039

## Data Availability

Available data on request.

## Supplementary Materials

The supporting information can be downloaded at: https://www.mdpi.com/article/10.3390/membranes15040111/s1. Figure S1. SEM images from membrane wall structure from M1 membrane at 1000×; Figure S2. SEM images from membrane wall structure from M1 membrane at 2500×; Figure S3. SEM images from membrane wall structure from M2 membrane at 1000×; Figure S4. SEM images from membrane wall structure from M2 membrane at 2500×; Figure S5. SEM images from membrane wall structure from M3 membrane at 1000×; Figure S6. SEM images from membrane wall structure from M3 membrane at 2500×.
