# Supplementary material for: Integration of Specific Aeration Demand (SAD) into Flux-Step Test for Submerged Membrane Bioreactor
Source: Membranes (Basel). 2025 Apr 3;15(4):111. doi: 10.3390/membranes15040111 (PMC12029511; doi:10.3390/membranes15040111)
Supplement: Supplementary file 1 [file membranes-15-00111-s001.zip › membranes-3476940-supplementary.pdf]

membranes

MDPI

Supplementary Material (SM)

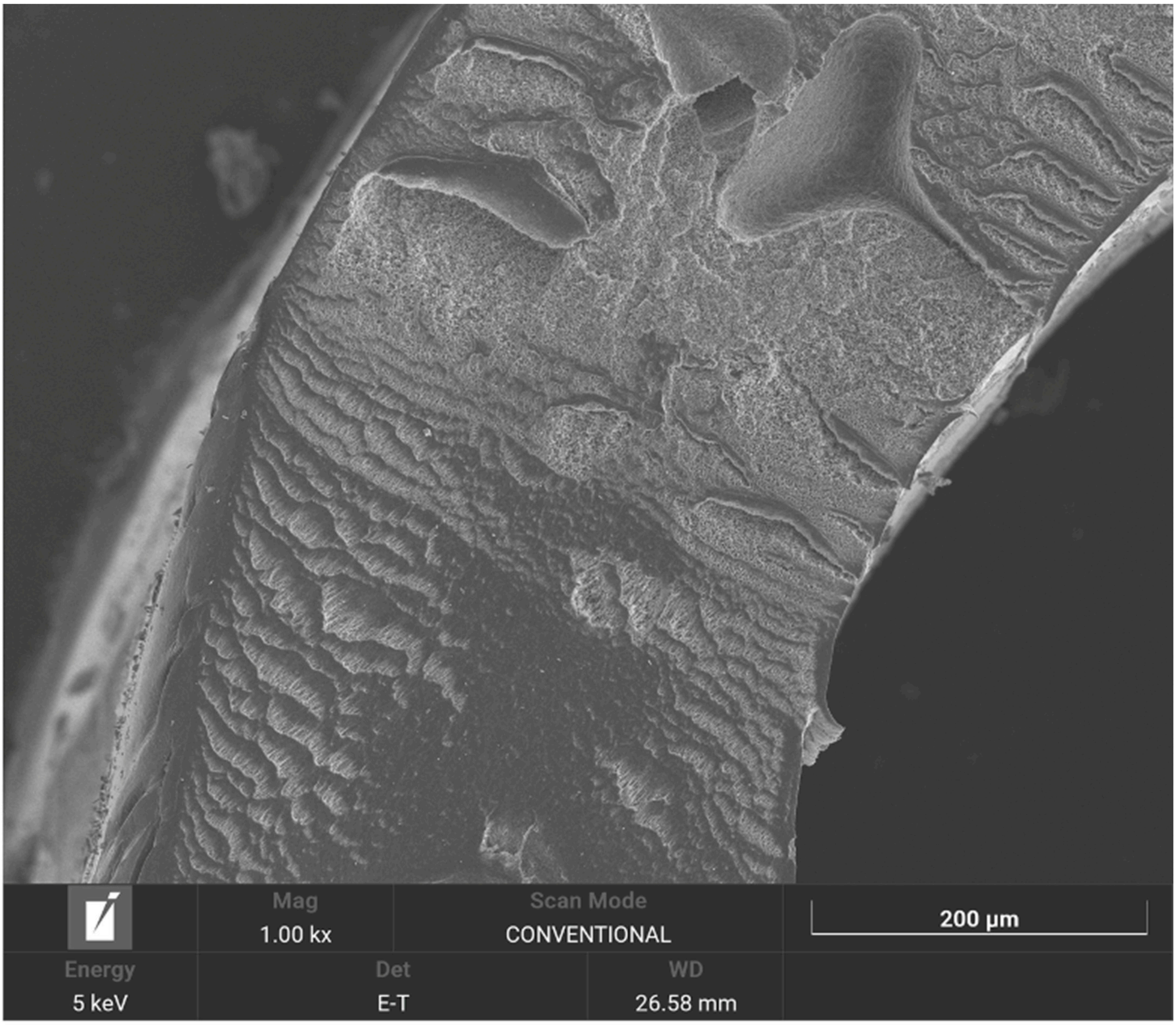

Figure S1. SEM images from membrane wall structure from M1 membrane at 1000×.

 https://doi.org/10.3390/membranes15040111

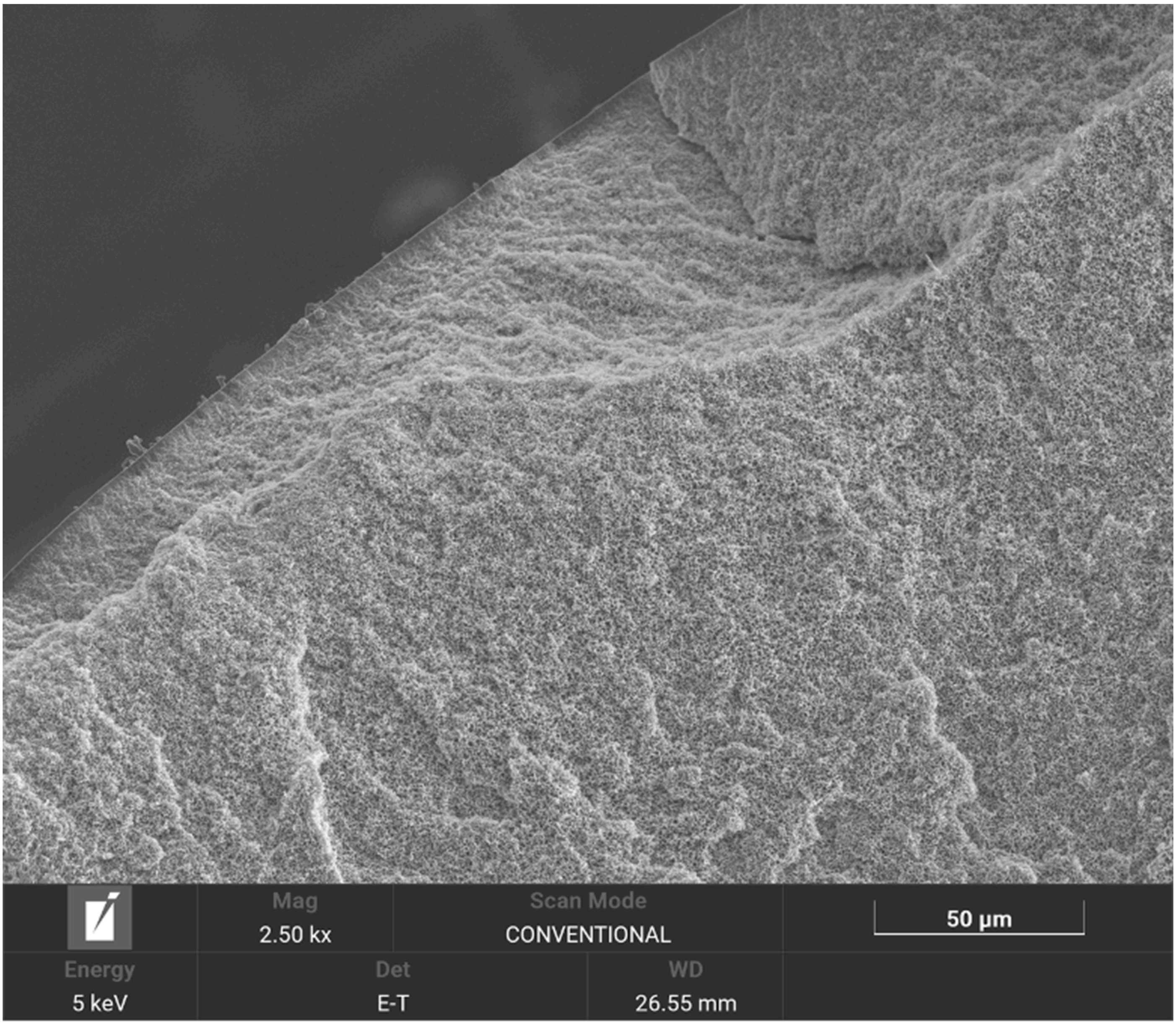

Figure S2. SEM images from membrane wall structure from M1 membrane at 2500×.

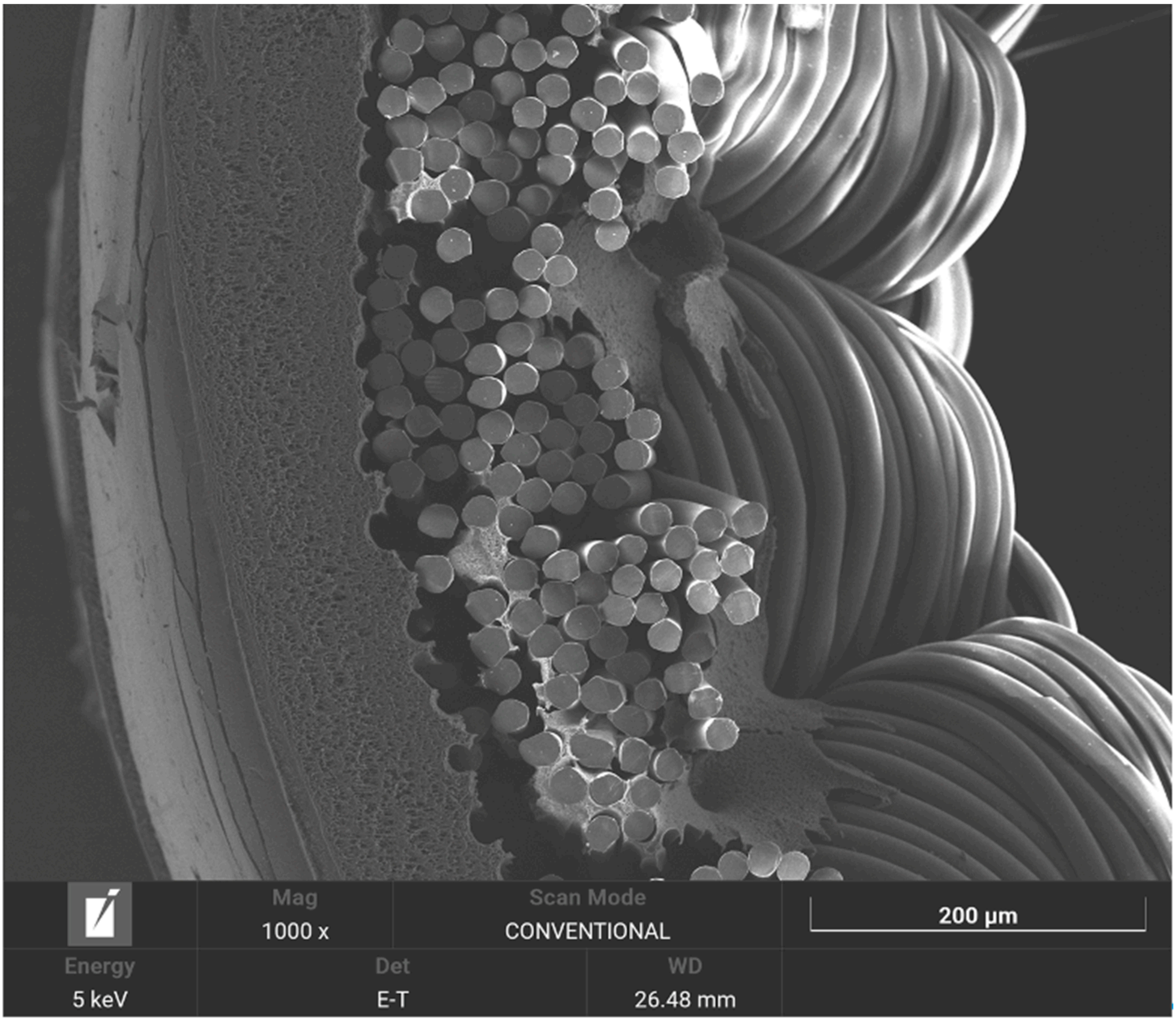

Figure S3. SEM images from membrane wall structure from M2 membrane at 1000×.

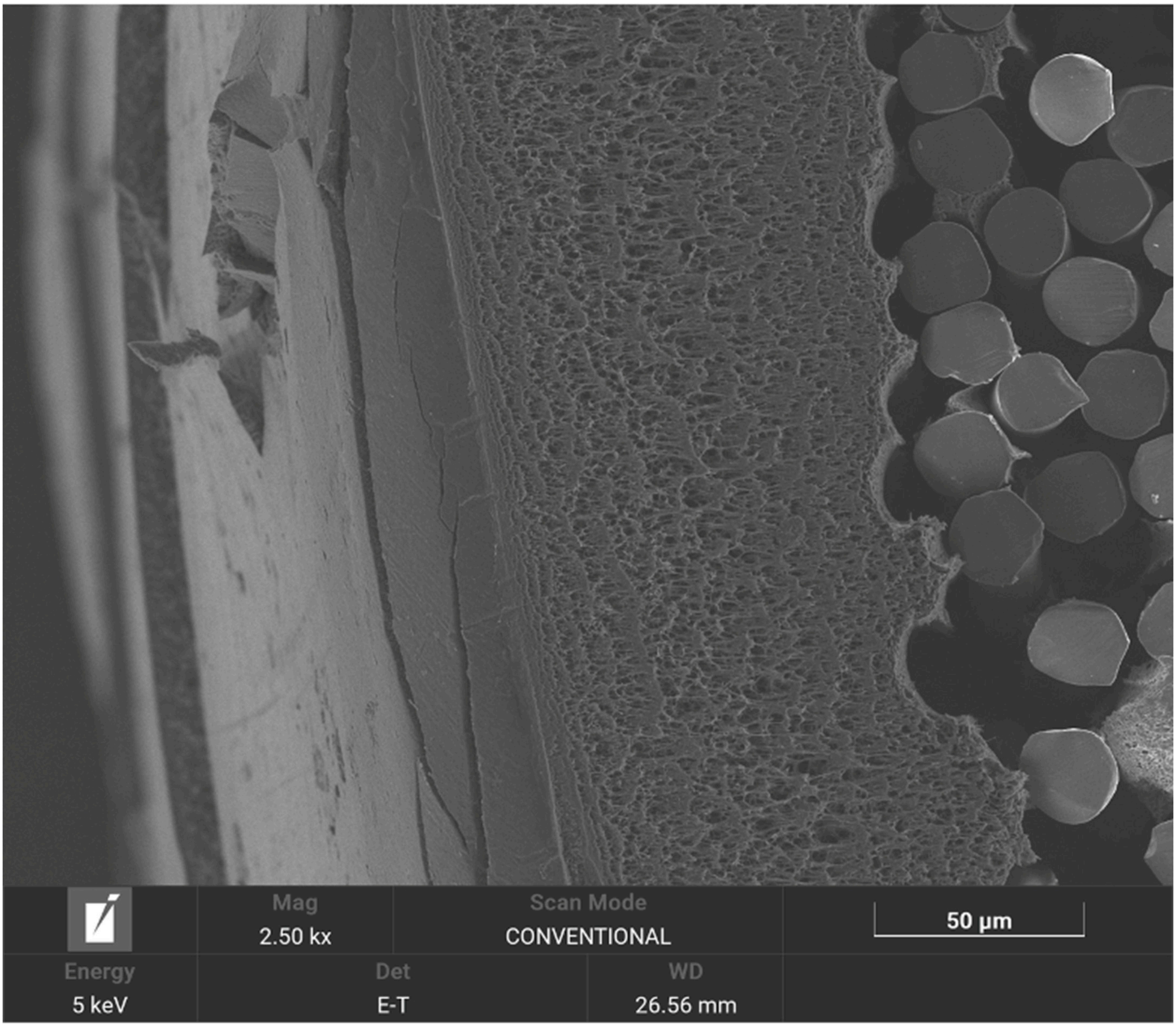

Figure S4. SEM images from membrane wall structure from M2 membrane at 2500×.

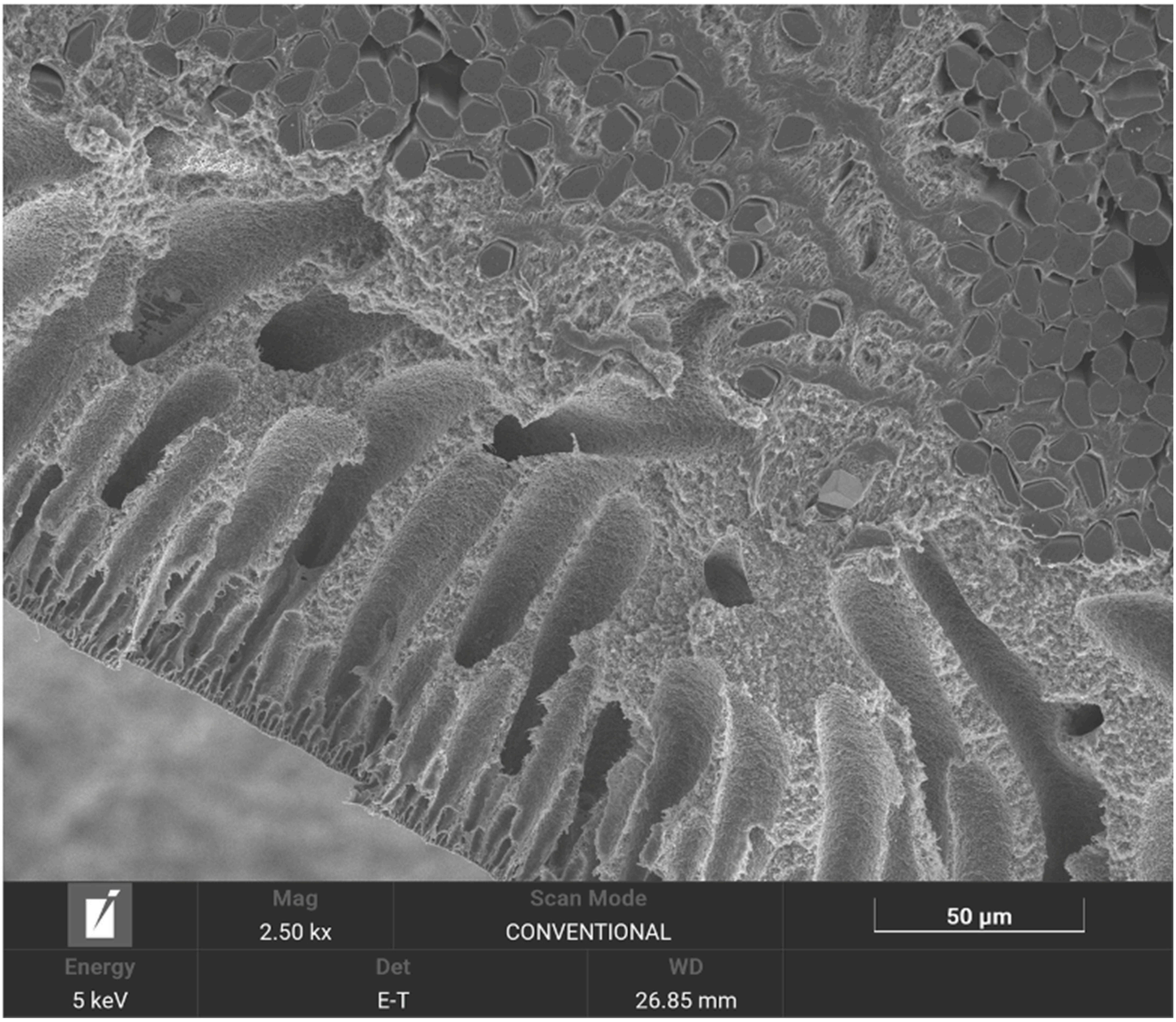

Figure S5. SEM images from membrane wall structure from M3 membrane at 1000×.

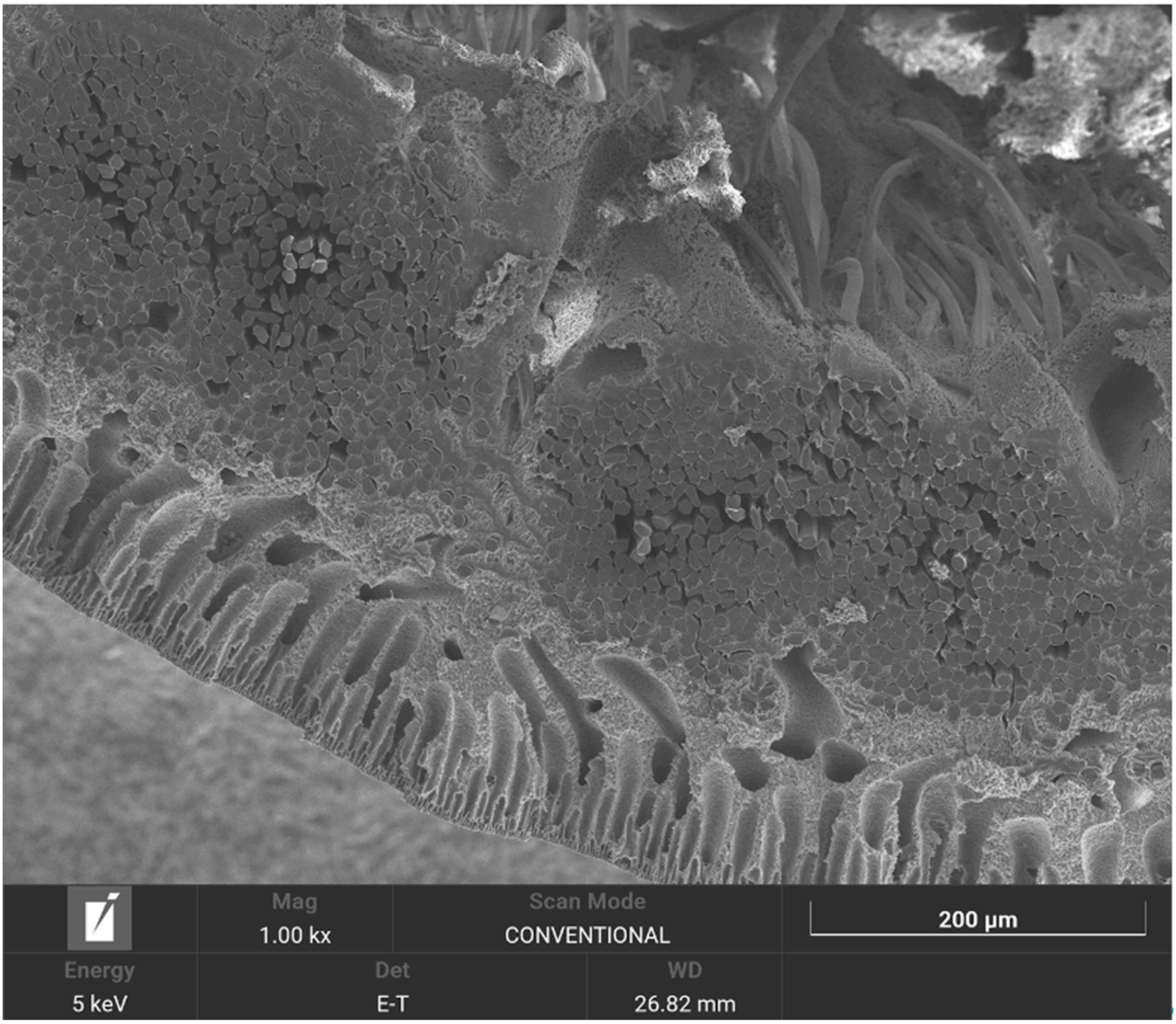

Figure S6. SEM images from membrane wall structure from M3 membrane at 2500×.
